# No associations exist between mean platelet volume or platelet distribution width and thyroid function in Chinese

**DOI:** 10.1097/MD.0000000000004573

**Published:** 2016-10-07

**Authors:** Xiaojun Ren, Zhaowei Meng, Ming Liu, Mei Zhu, Qing He, Qing Zhang, Li Liu, Kun Song, Qiyu Jia, Qiang Jia, Xue Li, Jian Tan, Wei Zheng, Renfei Wang, Na Liu, Tianpeng Hu

**Affiliations:** a Department of Endocrinology and Metabolism; b Department of Nuclear Medicine; c Department of Health Management, Tianjin Medical University General Hospital, Tianjin, P.R. China.

**Keywords:** mean platelet volume, platelet distribution width (PDW), sex, thyroid dysfunction, thyroid-stimulating hormone

## Abstract

Mean platelet volume (MPV) and platelet distribution width (PDW) are morphometric indices of size distribution and variability of platelet. We aimed to explore the associations between MPV or PDW and thyroid function in a large Chinese cohort.

This was a cross-sectional study with a recruitment of 13,622 self-reported healthy Chinese (8424 males, 5198 females). Clinical data of the participants comprised of anthropometric measurements, hepatic function, renal function, serum levels of lipid, glucose, C-reactive protein, erythrocyte sedimentation rate, platelet, MPV, PDW, and thyroid hormones. Database was sorted by sex, and the associations between MPV or PDW and thyroid function were analyzed by quartiles of MPV or PDW. Levels of MPV and PDW were compared in different thyroid function subgroups by 1-way analysis of variance and independent sample's *t* test. Receiver-operating characteristic (ROC) curve was adopted to determine diagnostic values of MPV and PDW for thyroid dysfunction. Crude and adjusted odds ratios of MPV and PDW for thyroid dysfunction with 95% confidence intervals were analyzed by binary logistic regression models.

MPV, PDW, and thyroid stimulation hormone were significantly higher in females than in males. Females showed significantly higher incidence of hypothyroidism and hyperthyroidism than males. However, there were no significant differences of MPV and PDW among different thyroid function subgroups in both sexes, and no obvious correlations were revealed between MPV or PDW and thyroid function. From ROC analysis, we demonstrated no diagnostic values of MPV and PDW for thyroid dysfunction. From binary logistic regression models, no risks of different MPV and PDW quartiles were identified for thyroid dysfunction in both sexes.

We could not show any association between MPV or PDW and thyroid function. Prospective studies with better defined risk groups should be performed in the future for further verification and validation.

## 1 Introduction

Platelets (PLT) play a central role not only in hemostasis and thrombosis, but also in inflammation, atherogenesis, and even cancer.^[1]^ Mean platelet volume (MPV) and platelet distribution width (PDW) are morphometric indices as well as quantitative measures of size distribution and variability of PLT, which can be called as platelet volume index (PVI) collectively. MPV and PDW are considered as integral components in the general assessment of PLT function.^[2]^ PDW and MPV have been reported to be associated with a wide variety of diseases; however, there are obvious contradictions in the literatures. For instance, some investigations found MPV was positively correlated with the presence of coronary artery atherosclerosis,^[3]^ degree of coronary artery calcification,^[4]^ and cardiovascular mortality.^[5]^ Yet, other reports did not identify any association between MPV and the extent of coronary artery disease.^[6,7]^ Park et al^[8]^ showed that patients with metabolic syndrome had significantly lower MPV levels, which phenomenon was independent of confounding variables only in women. However, Lippi et al^[9]^ found that MPV in patients with metabolic syndrome was not significantly different from the control subjects. Surprisingly, Demirtunc et al^[10]^ even displayed that patients with the metabolic syndrome had a significantly higher MPV compared with the control group. MPV was demonstrated to correlate with type 2 diabetes mellitus,^[11]^ but among diabetic patients, conflicting results were reported between MPV and hemoglobin A1C levels, or micro- or macrovascular complications.^[11–13]^ Likewise, there were also inconsistent reports on the role of MPV for predicting atrial fibrillation-related illness,^[14,15]^ as well as ischemic stroke.^[16–18]^

The relationship between MPV or PDW and thyroid has not been comprehensively studied. Moreover, the available published literatures are far from conclusive. For example, several investigations revealed increased MPV in subclinical hypothyroidism,^[19–22]^ Hashimoto thyroiditis^[23]^ or hyperthyroidism,^[24]^ but other studies did not demonstrate such phenomenon.^[25–27]^ Very recently, Lippi et al^[28]^ studied 1050 ostensibly healthy and euthyroid patients aged 50 years and older, and demonstrated a significant, positive, and independent association between MPV and serum thyroid-stimulating hormone (TSH) values. Thyroid diseases are very common, particularly in women. The prevalence of hypothyroidism and hyperthyroidism has been reported as approximately 8.9%^[29]^ and 1.2%.^[30]^ It has been reported that coagulation-fibrinolytic system is very sensitive to thyroid hormones.^[31]^ Therefore, the open question of whether MPV or PDW has any diagnostic or predictive values for thyroid dysfunction still awaits definite answers.

In this study, we aimed to systematically investigate the associations between MPV or PDW and thyroid function in a cohort of Chinese.

## 2 Methods

### 2.1 Design

Since 2007, we commenced a cross-sectional, community-based health-check program in Tianjin Medical University General Hospital, with the joint efforts form departments of Health Management, Endocrinology & Metabolism, and Nuclear Medicine. Our first publication was released in 2011,^[32]^ and now we had a total of 5 publications on this continuous project,^[32–36]^ from which investigational methodology was described in details. Briefly, all of the self-reported healthy participants were asked to fill in a questionnaire, and then blood samples were obtained. From September 2011, because of the allowance of our budget, thyroid hormone level test was included in the research. For the purpose of the present study, to avoid the influences of confounding factors, the followings were set as the exclusion criteria: subjects with known histories of liver, kidney, gastrointestine, cardiovascular, inflammation, infection, immune, oncology, thyroid, or hematological diseases, diabetes; participants taking any medicine (such as antithrombotics, aspirin, antilipemics, antihypertensives, diuretics, oral contraceptives, among others) that might influence hematology, thyroid, inflammation, infection or immune; pregnancy. A total number of 13,622 eligible Chinese (8424 males, 5198 females) with adequate data for analysis were included in this research. They were recruited during the time period from September 2011 through March 2014. Written consent was provided from every person of the recruits, and the institutional review board and ethic committee of Tianjin Medical University General Hospital approved this study.

### 2.2 Sample and measurement

Fasting blood tests, as well as anthropometric measurements, were done when the participants visited our institution. Body height (BH) and body weight (BW) were measured with light indoor clothing and without shoes, and body mass index (BMI) was calculated by using the equation “BW (kilograms) divided by the square of BH (meters^2^)”. Peripheral venous blood samples were collected for the following test items: alanine aminotransferase (ALT), total bilirubin (TBIL), blood urea nitrogen (BUN), creatinine (Cr), total cholesterol (TC), triglycerides (TG), and fasting glucose (FG) by an automated analyzer (Hitachi Corporation, Tokyo, Japan); C-reactive protein (CRP) on an analyzer (Hebai Diagnostics, Shijiazhuang, China); erythrocyte sedimentation rate (ESR) by Westergren method (Yakun Diagnostics, Tianjin, China); TSH, free triiodothyronine (FT3), and free thyroxine (FT4) by chemiluminescent reaction principle on an automated ADVIA Centaur analyzer (Siemens Healthcare Diagnostics, Erlangen, Germany).

For the measurement of PLT, MPV, and PDW, in specific, venous blood samples were obtained and collected in K2-ethylenediaminetetraacetic acid (EDTA) tubes. The PLT count, MPV, and PDW values were analyzed on an automated hematological analyzer (Sysmex Corporation, Kobe, Japan) within 60 minutes of sample collection.

The laboratory calibration references for the above parameters were as follows: ALT 5 to 40 U/L; TBIL 3.4 to 20 μmol/L; BUN 1.7 to 8.3 mmol/L; Cr 44 to 115 μmol/L; TC 3.5 to –5.17 mmol/L; TG 0.57 to 1.71 mmol/L; FG 3.6 to 5.8 mmol/L; CRP <8 mg/L; ESR <20 mm/h; TSH 0.3 to 5.0 mIU/L; FT3 3.5 to 6.5 pmol/L; FT4 11.5 to 23.5 pmol/L; PLT 125 to 350 × 10^9^ cells/L; MPV 7.8 to 12.5 fL; PDW 9.0 to 17.0 fL.

### 2.3 Grouping and definition

According to the TSH reference, thyroid function subgroups were determined. Hypothyroidism was determined as TSH >5.0 mIU/L, hyperthyroidism as TSH <0.3 mIU/L, and euthyroidism as 0.3 ≤ TSH ≤ 5.0 mIU/L. MPV and PDW were divided according to the quartiles of their measurements. Age subgroups 1 to 6 were defined as the followings: age ≤25 years, 25 years < age ≤ 35 years, 35 years < age ≤ 45 years, 45 years < age ≤ 55 years, 55 years < age ≤ 65 years, age > 65 years.

### 2.4 Statistical analysis

All data were presented as mean ± standard deviation (SD). Differences of the parameters between groups or subgroups were measured by independent sample *t* test or 1-way analysis of variance. Intergroup or intersubgroup prevalence differences were compared by *χ*^2^ test. Pearson bivariate correlation was conducted among different variables. Diagnostic values of MPV and PDW for thyroid dysfunction were performed by using receiver-operating characteristic (ROC) curve. Crude and adjusted odds ratios (ORs) for thyroid dysfunction with 95% confidence intervals (CIs) were analyzed by binary logistic regression models. We adopted the software of Statistical Package for Social Sciences (SPSS version 17.0, Chicago, IL) for all statistical analyses. Significance was defined as *P* < 0.05.

## 3 Results

### 3.1 Characteristics of the participants

**Figure 1:**
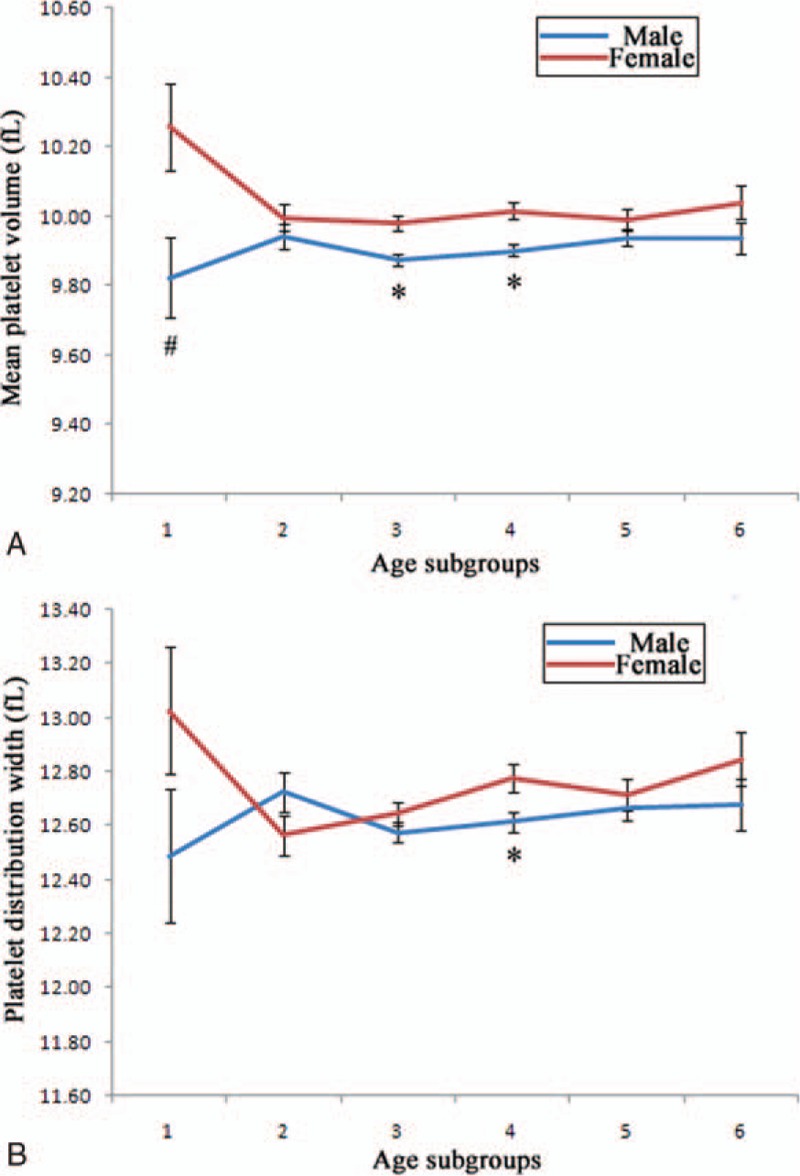
Mean platelet volume and platelet distribution width in different age subgroups. All participants were analyzed for mean platelet volume (A) and platelet distribution width (B) by different sex separately. Subgroups 1 to 6 referred to the followings ages respectively: age = 25 years, 25 years < age = 35 years, 35 years < age = 45 years, 45 years < age = 55 years, 55 years < age = 65 years, age > 65 years. ^#^Difference of prevalence between sex was significant at 0.05; ^∗^difference of prevalence between sex was significant at 0.01.

There were significant differences in all indices among opposite sex (Table 1). Females were older than males. TC, ESR, PLT, MPV, PDW, and TSH were significantly higher in females than in males. However, all the other parameters were significantly higher in males than in females. Females showed decreased levels of MPV and PDW from the youngest age subgroup to the age subgroup between 25 and 35 years, whereas males showed increased patterns of them. After 35 years of age, MPV and PDW remained relatively constant among different age subgroups, especially for MPV (Fig. 1). Generally speaking, females demonstrated higher levels of MPV and PDW than males. In particular, significant differences existed for the age subgroup between 45 to 55 years for both MPV and PDW.

**Table 1:**
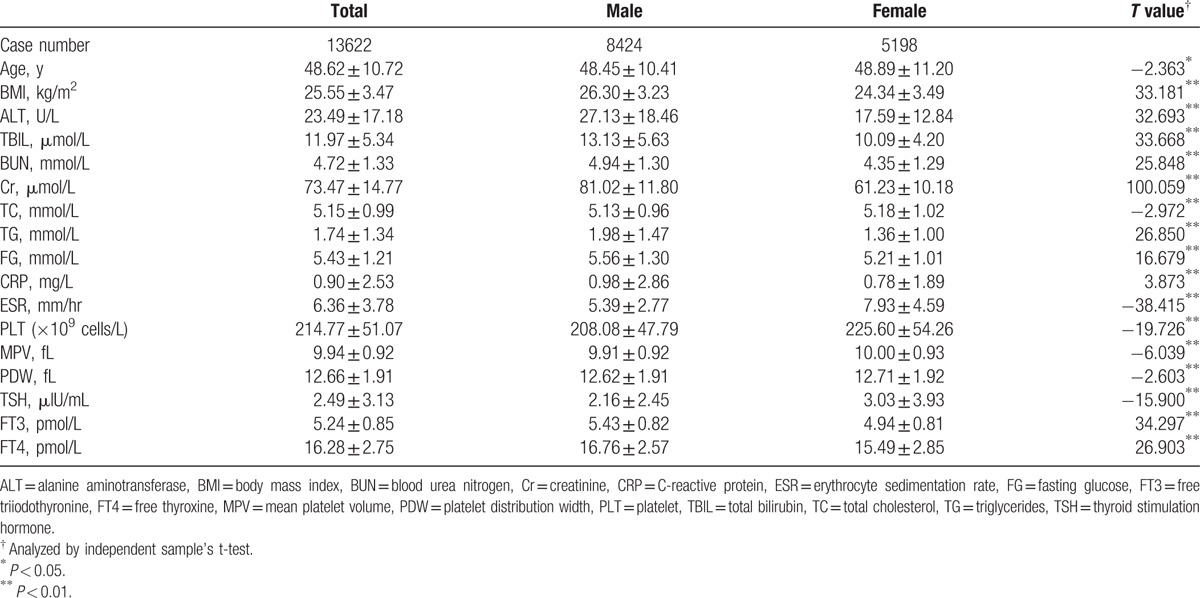
Population characteristics.

### 3.2 Incidence of thyroid dysfunction

Females demonstrated significantly higher overall incidences of hypothyroidism and hyperthyroidism than males. Subgroup incidences of hypothyroidism and hyperthyroidism (divided by MPV and PDW quartiles) were analyzed separately; all subgroups displayed the same differences between women and men (Tables 2 and 3).

**Table 2:**
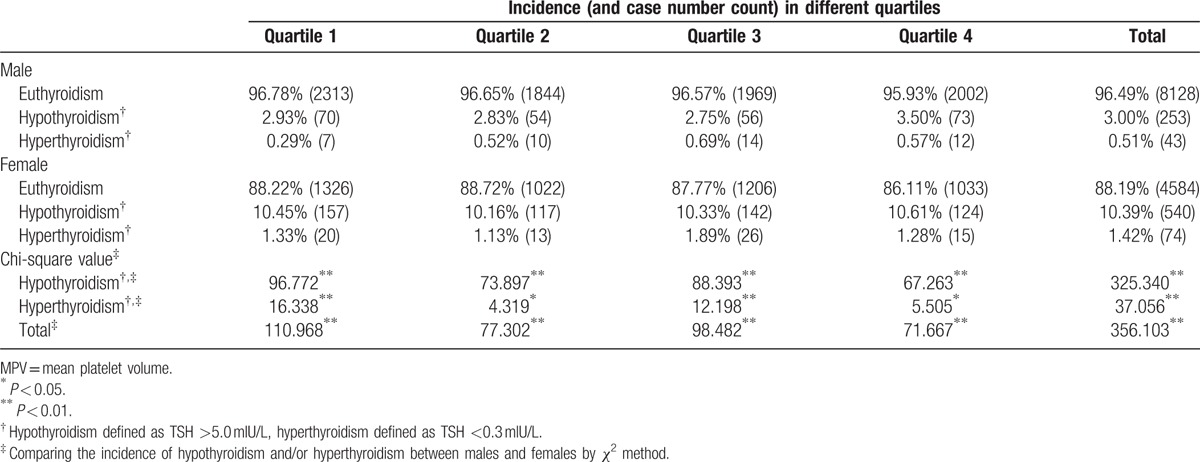
Incidence of thyroid dysfunction in different sexes by MPV quartiles.

**Table 3:**
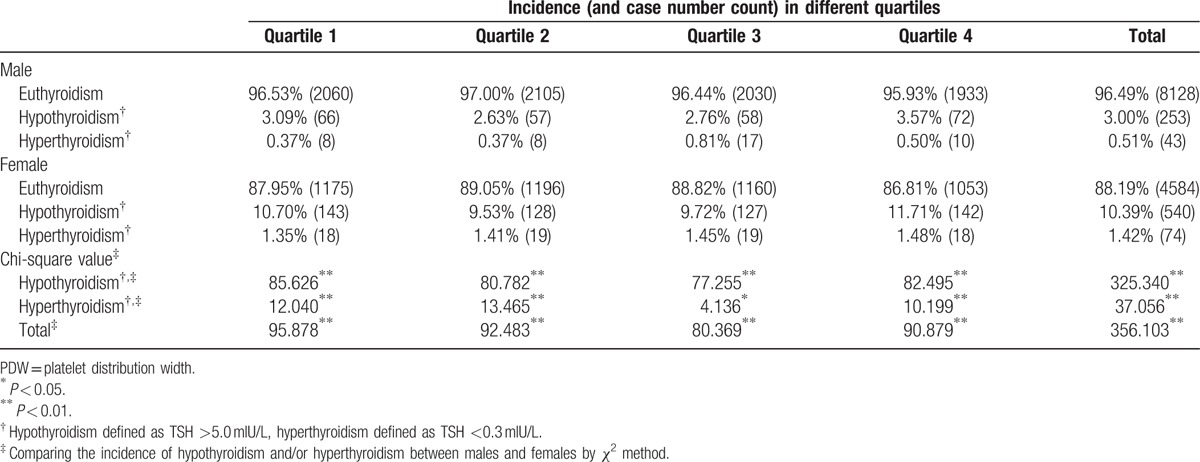
Incidence of thyroid dysfunction in different sexes by PDW quartiles.

### 3.3 MPV and PDW levels in different thyroid function subgroups

Levels of MPV and PDW were compared among different thyroid function subgroups in different sex respectively. No significant differences were identified in the parameters (Table 4).

**Table 4:**
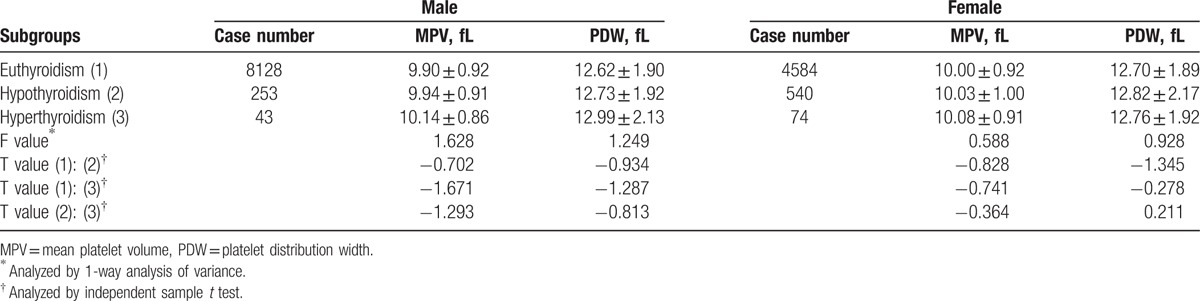
MPV and PDW levels in different thyroid function subgroups.

### 3.4 Correlations of MPV and PDW with other indices

For men, both MPV and PDW demonstrated significant positive relationships with BMI, hepatic function, renal function, and FG, yet negative relationships with TC, ESR, and PLT. For women, both MPV and PDW displayed significant positive relationships with TBIL only, but negative relationships with BMI, TC, CRP, and PLT. For both sexes, no obvious correlations were identified between MPV or PDW and age or thyroid function (Table 5).

**Table 5:**
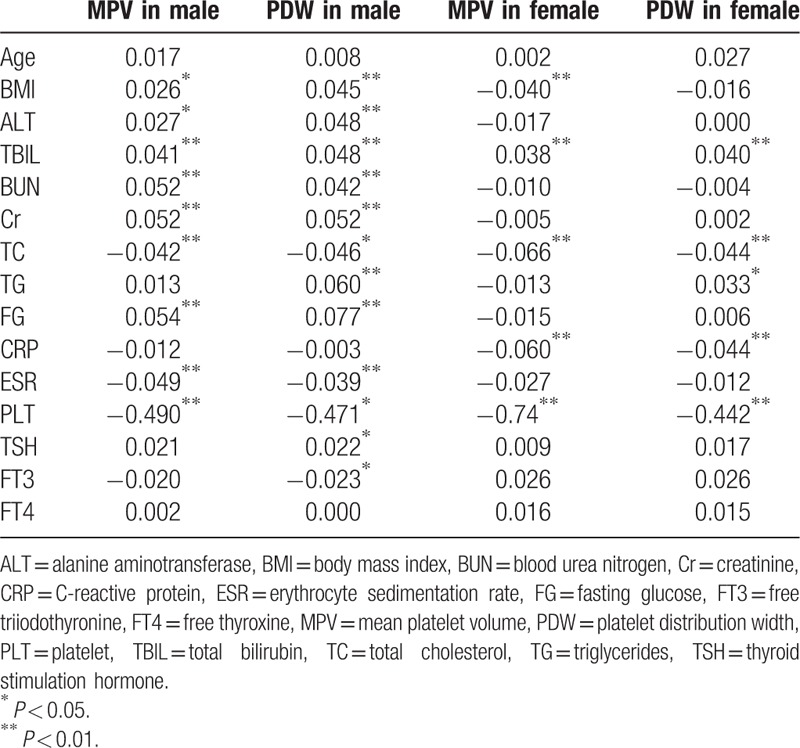
Pearson bivariate correlation coefficients.

### 3.5 Diagnostic values of MPV and PDW for thyroid dysfunction

From ROC analyses, MPV and PDW demonstrated no diagnostic and predictive values for either hypothyroidism or hyperthyroidism. Areas under the curves of MPV and PDW were found to be 0.520 and 0.518 in males for hypothyroidism (both *P* > 0.05), 0.580 and 0.549 in males for hyperthyroidism (both *P* > 0.05), 0.505 and 0.508 in females for hypothyroidism (both *P* > 0.05), and 0.526 and 0.512 in females for hyperthyroidism (both *P* > 0.05).

### 3.6 Risks of thyroid dysfunction in different MPV and PDW quartiles

Risks of thyroid dysfunction in different sexes were calculated by utilizing binary logistic regression models (Table 6). Crude OR calculation was performed with lowest quartiles of MPV and PDW as references. Adjusted risk factors included age, BMI, ALT, TBIL, BUN, Cr, TC, TG, FG, CRP, ESR, PLT, FT3, and FT4 as covariates. We could not identify any significant risk for thyroid dysfunction in both males and females (*P* > 0.05).

**Table 6:**
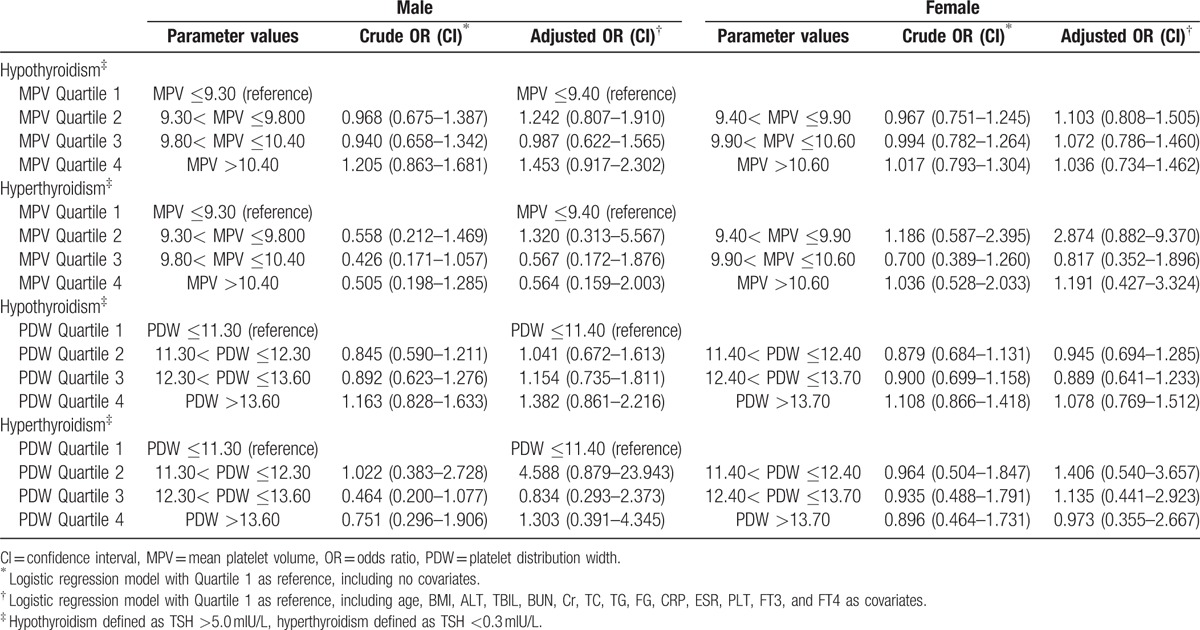
The risks of thyroid dysfunction according to MPV and PDW quartiles.

## 4 Discussion

PVIs are a group of parameters derived from routine blood counts. MPV and PDW are the most validated representatives of PVIs. MPV and PDW are not only inexpensive but also universally available with blood routine measurements, which can be automatically calculated from PLT count by hematology analyzers. MPV (measured in femtoliters) can be deduced from the following formula,^[2,37]^ in which the plateletcrit represents the ratio of PLT volume to whole blood volume: MPV = (plateletcrit [%]/PLT count [10^9^ cells/L]) × 10^5^. Other PVIs, such as PDW, can be derived from the PLT size distribution curve. The distribution width at the level of 20% is defined as PDW. PVIs are traditionally considered useful in assessing the etiology of thrombocytopenia.^[38]^ For example, MPV and PDW can be utilized to differentiate between hypoproductive thrombocytopenia (e.g., aplasia anemia) and hyperdestructive thrombocytopenia (e.g., idiopathic thrombocytopenic purpura).^[39]^

There are an increasing number of studies evaluating diagnostic and prognostic values of PVIs, particularly MPV, in diseases other than hematology. However, obvious inconsistence exists. In cardiovascular diseases, for instance, Jung et al^[4]^ found that patients with coronary artery calcification had significantly higher MPV levels than otherwise, and regarded MPV as an independent predictor of coronary artery calcification. In an Austria study conducted by Slavka et al,^[5]^ a total of 206,554 first-ever admissions to the Allgemeines Krankenhaus Wien were included between January 1996 and July 2003. Patients with increased MPV levels (≥11.01 fL) were found at higher risks of death because of ischemic heart disease. The authors proposed that increased MPV contributes to the phenomenon by the following mechanisms^[4,5]^: larger PLT was both metabolically and enzymatically more active than smaller ones, containing more prothrombotic materials (e.g., thromboxane A_2_ and B_2_) and increased levels of procoagulant surface proteins; larger PLT had more α-granules, containing chemotactic and mitogenic factors that could cause vascular intimal proliferation; during the formation of an arteriosclerotic plaque, PLT played an essential role during the subsequent thrombus assembly, leading to myocardial infarction, whereas MPV was a simple and accurate marker of the functional status of the process. On the contrary, De Luca et al^[6]^ showed that even combined information on MPV and PDW was not related to the extent of coronary artery disease. The authors gave an explanation for such a negative result.^[6]^ They considered that increase in PLT volume and its variability might be a process driven by increased production of reticulated PLT from bone marrow. This could be because of the compensatory effect of consumption of small PLT in coronary artery disease.^[7]^ Thereafter, larger PLT volume and its larger variability might not imply higher reactivity, but an indication of even reduced aggregation as larger PLT could be precursor and not fully mature PLT.

In the case of MPV with metabolic syndrome, conflicting results were also reported. In a study form Korea, Park et al^[8]^ recruited 3827 participants who voluntarily underwent medical examinations, and found that female subjects with metabolic syndrome had significantly lower MPV than otherwise. The mechanism was proposed as followed^[8]^: increased adiposity tissue in metabolic syndrome could engender increasing secretions of adipokines and cytokines such as leptin, adiponectin, interleukin 6, and tumor necrosis factor α, which could lead to chronic low-grade inflammation and increase PLT counts. And since MPV was usually inversely associated with PLT counts (also demonstrated in Table 5 of the present study), so that the PLT mass (PLT count × MPV) could be kept constant. MPV was inversely related with metabolic syndrome as a result. Nevertheless, the investigation conducted in Italy by Lippi et al,^[9]^ which enrolled 3337 participants, only found slightly higher level of MPV in subjects with metabolic syndrome than otherwise, but there was no significant difference. The completely contrary findings were demonstrated in a prospective, randomized Turkish study from Demirtunc et al^[10]^ in which patients with the metabolic syndrome had a significantly higher MPV compared with the control group, and MPV was significantly decreased after doxazosin treatment. They reasoned that as a key mechanism for metabolic syndrome, insulin resistance was associated with PLT aggregability and reduced prostacyclin generation, which resulted in enhanced PLT activity and increased MPV level.

The present study focused on the relationship between PVI and thyroid, with the largest sample size for the topic until now. We did not identify any associations between MPV or PDW and thyroid function. Confusing literature in this particular topic can also be retrieved. Three studies from Turkey^[19–21]^ and 1 study from Korea^[22]^ showed increased MPV levels in subclinical hypothyroidism, and another Turkish research^[23]^ showed elevated MPV in Hashimoto's thyroiditis. The sample sizes of all the Turkish studies^[19–21,23]^ were very small (<100 cases in the patients group). An Italian cohort consisting of 1050 ostensibly healthy and euthyroid patients aged 50 years and older showed a graded increase of MPV values from the first to the fourth quartile of TSH.^[28]^ The authors proposed that elevated TSH had a prothrombotic effect, leading to cardiovascular consequences (like atherosclerosis), and MPV was a marker of such adverse events. And it was suggested that PLT activation, as reflected by an increased MPV, might be an important mediator of thrombotic complications in patients with fluctuations of thyroid hormones. Nevertheless, an early small-sample study from the Netherlands^[25]^ showed hypothyroidism could lead to more small-sized PLT, and a decrease of MPV. Another recent small-sized investigation from Turkey^[26]^ also did not observe significant MPV change in subclinical hypothyroidism. Torun et al^[26]^ explained that increase of MPV was owing to several inflammatory cytokines, and the effect of hypothyroidism on MPV seemed unlikely without an additional risk factor affecting low-grade inflammation. We also agree with the comments and suggestions from Varol^[40]^ on the article of Carlioglu et al^[23]^ that various confounding factors (e.g., obesity, hepertension, smoking, hyperlipidemia, atrial fibrillation, rheumatic diseases, inflammatory diseases, metabolic syndrome, diabetes mellitus, among others), which can affect MPV values, should be taken into consideration when studying MPV. In our research, we could not prove any existed correlations between MPV or PDW and thyroid with or without various factors in our regression models (Table 6).

The measuring technique of MPV should be considered seriously when conducting such investigations because accurate measurement of MPV is important for clinical and research purposes. However, there are several crucial factors that can influence the results. Generally, variation and discordance of MPV are implicated in the following major confounding factors: anticoagulant type (EDTA or citrate), time interval between blood sampling and MPV analysis, and environmental temperature during storage and measurement.^[41–45]^ PLT swells in EDTA, but shrinks in citrate.^[2,44]^ However, it is mandatory that blood samples be anticoagulated to inhibit coagulation before measurement. According to the recommendation from International Council for Standardization in Hematology,^[46]^ most laboratories, like ours, use EDTA for anticoagulation. For instance, in the 10 articles that we could retrieve studying PVI and thyroid, 5 used EDTA,^[19,22,24,25,28]^ 4 used citrate,^[20,21,26,27]^ and 1 did not contain such information.^[23]^ Dastjerdi et al^[41]^ proposed measuring time should be within 60 minutes of sampling for EDTA, whereas Lancé et al^[43]^ showed the time delay should be within 120 minutes for EDTA and 60 minutes for citrate. Analysis of blood samples within 120 minutes of venipuncture was also recommended by Leader et al.^[2]^ Nowadays, it is widely accepted that PLT swelling in EDTA tubes can be minimized by rapid analysis of samples (within 60 minutes).^[40,42]^ In daily practice, MPV measurements are performed at room temperature, so this factor can be negligible. In the current investigation, we adhered to the optimal preanalytical methodologies for PVI determination. Still the urgent matter is, since the study on MPV is becoming increasingly popular, a measurement standard or consensus should be developed and implemented as soon as possible.

Besides preanalytical condition and measuring techniques, there are a large number of genetic and nongenetic determinants for MPV.^[44]^ Panova-Noeva et al^[44]^ displayed that age, cardiovascular risk factors, hypertension, and hyperglycemia were linked with higher MPV in males. Intake of oral contraceptives and menstruation were strongly associated with higher MPV in females. Moreover, 7 single-nucleotide polymorphisms (rs342293, rs7961894, rs12485738, rs649729, rs342251, rs17568628, rs4774471) for females and 4 single-nucleotide polymorphisms (rs342293, rs7961894, rs10876550, rs342251) for males were associated with higher MPV. In addition, Leader et al^[2]^ demonstrated that MPV was not only involved in risk factors of cardiovascular disease, but also in gestational diabetes, pre-eclampsia, and various kinds of inflammatory diseases. Thereby, for accurate MPV measurement, all the above factors should be taken into account. Our investigation was based on a cross-sectional heath-check program, not just a PVI or thyroid-oriented study. Although we tried to limit confounding factors, but actually, we could not encompass all parameters, especially the single-nucleotide polymorphisms traits of the participants. This is an intrinsic limitation for our research.

There are several limitations of our study we want to elaborate. And at the same time, we also intend to share some thoughts on this subject at this stage, which could hopefully motivate future researches. First, as explained above, it is not suitable to compare results from different measurement methodologies (e.g., the usage of anticoagulant EDTA versus citrate will engender different results). Future investigations should comply with a standard method, which should include recommended anticoagulant type, time delay from sampling to measurement, technique implemented, and optimal temperature. Second, as MPV is affected by a myriad of factors, it is not feasible to study a larger group of participants than the current one. The larger study group, the larger heterogeneity of the population, and the more regression to the mean we will get, let alone conducting the research in a reasonable amount of time. Therefore, we would rather suggest doing smaller studies in better-defined risk groups and see whether this relationship becomes apparent in the future. Third, the cross-sectional nature of the present study is another inherent shortcoming. Prospective investigations should be conducted in the future. Fourth, we did not ask the participants about specific thyroid dysfunction symptoms (e.g., obvious fatigue, weight gain, laziness, edema, constipation for hypothyroidism) because the study was a general health check-up, not a thyroid solely oriented questionnaire. This is another shortcoming of the present study. Fifth, we did not measure cytokines, interleukins, adipokines, thyroid antibodies, and sex hormones in the investigation owing to budget shortage. Inflammation, in specific inflammatory markers, has been implied to play a certain role in the pathogenesis of both MPV^[2,47,48]^ and thyroid diseases.^[49,50]^ If budget allows, it would be interesting to measure these markers to see whether or not these markers play a role in establishing a link between PVI and thyroid. Or perhaps, these markers are just some bystanders. In fact, it has been demonstrated that inflammation-related cytokines and adipokines could be the underlying mechanism between PVI and metabolic syndrome.^[8]^ Sixth, we applied strict exclusion criteria to rule out confounding factors, yet a number of participants with disease conditions might not be aware of their medical status, which could also be a disadvantage in our research. Future investigations should implement more rigorous group stratifying method. Perhaps a preliminary medical checking, including single-nucleotide polymorphisms traits determination, should be conducted before formal recruitment, to minimize confounding factors.

## 5 Conclusion

The present study could not identify any associations between MPV or PDW and thyroid function. Our findings question the utility of MPV or PDW for differential diagnosis of thyroid dysfunction, the relationship of which should be considered with caution in clinical practice. Future verification studies should be conducted in better-defined risk groups with smaller size, ideally in prospective nature, to determine whether the relationships become apparent.

## Acknowledgments

Many thanks for the statistical consultation from Dr. Ping-Shou Zhong and Dr. Heng Wang (Department of Statistics and Probability, Michigan State University), who helped us review the statistics of the manuscript.
